# Prolactin-producing pituitary adenoma with atypical spindle cell morphology: a case report

**DOI:** 10.1186/s12957-015-0655-x

**Published:** 2015-07-31

**Authors:** Ritsurou Inoue, Mikiko Aoki, Yoshihisa Matsumoto, Seiji Haraoka, Kiyoshi Kazekawa, Kazuki Nabeshima

**Affiliations:** Department of Neurosurgery, Fukuoka University Chikushi Hospital, 1-1-1 Zokumyoin, Chikushino, Fukuoka 818-8502 Japan; Department of Pathology, School of Medicine, Fukuoka University, 7-45-1 Nanakuma, Jonan-ku, Fukuoka 814-0180 Japan; Department of Pathology, Fukuoka University Chikushi Hospital, 1-1-1 Zokumyoin, Chikushino, Fukuoka 818-8502 Japan

**Keywords:** Pituitary adenoma, Prolactin, Morphologic atypia, Spindle cell

## Abstract

Reported herein is a 25-year-old woman who was treated for a large and highly atypical prolactin-producing pituitary adenoma. On presentation, she exhibited right hemiparesis and left-sided visual loss, associated with amenorrhea. A massive (>5 cm) intra- and suprasellar lesion was seen on imaging, and her serum prolactin level was 4408 ng/ml. The patient received dopamine agonist treatment preoperatively for 4 weeks. To resect the tumor, a two-stage excision was required. Histologically, the specimen was composed of polygonal or spindle cells showing marked nuclear pleomorphism and/or multinucleation. Fibrosis was also focally conspicuous. Differential diagnoses included pituitary adenoma, pituitary carcinoma, pituicytoma, paraganglioma, spindle cell oncocytoma, and meningioma. Immunohistochemically, the tumor cells were positive for prolactin, chromogranin-A, and synaptophysin, but were negative for glial fibrillary acidic protein, S-100 protein, epithelial membrane antigen, and vimentin. No apparent cerebrospinal or systemic metastases are found. Ultimately, prolactin-producing pituitary adenoma was diagnosed. Our case highlights the difficulty in definitively diagnosing an unusual prolactin-producing adenoma based on histopathology alone and the importance of referring to clinical information and immunohistochemical findings when deriving the diagnosis.

## Background

In tissue sections, prolactin (PRL)-producing pituitary adenomas typically present as monotonous arrays of chromophobic cells in diffuse, papillary, or sinusoidal proliferations. Such cells generally are of uniform size, lacking both nuclear atypia and mitotic figures. Cystic change, bleeding, or calcification may be observed in the event of pituitary apoplexy. Likewise, dopamine agonist therapy may alter tumor histology, resulting in negative immunoreactivity for PRL; cellular shrinkage, degeneration, or necrosis; acellular or fibrotic areas; and other effects [1–3].

In this report, an unusual occurrence of prolactinoma is detailed. As opposed to the ordinarily bland picture of such lesions, the alarming degree of atypia we observed raised suspicions of pituitary carcinoma, paraganglioma, and pituicytoma. The histologic attributes of this particular tumor are discussed herein, supplemented by findings in prior publications.

## Case presentation

**Fig. 1:**
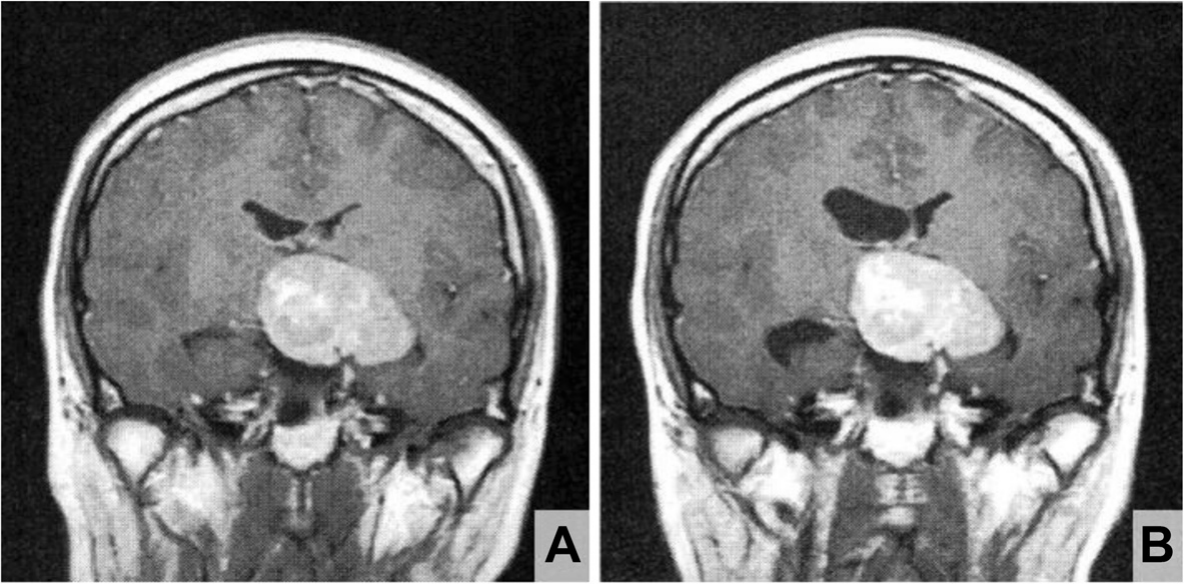
Pretreatment (**a**) and preoperative (**b**) coronal T1-weighted magnetic resonance images with contrast (gadolinium). Prior to surgery, slight dilatation of right lateral ventricle evident (**b**), compared with baseline status (before cabergoline therapy) (**a**)

A 25-year-old female patient had noticed left-sided visual loss and amenorrhea 2 years prior to being seen, with right hemiparesis developing 6 months beforehand. Aggravated symptoms (drooping at corner of right lip and bilateral temporal hemianopia) finally prompted hospitalization. At that time, a massive (>5 cm) tumor of intra- and suprasellar location, excluding mesencephalon backward, was seen on computerized tomography (CT) of the head (Fig. 1a). Further testing revealed an exceedingly high-serum level of prolactin (PRL) (4408 ng/ml), whereas levels of all other pituitary hormones were within reference ranges (growth hormone (GH), 0.50 ng/ml; luteinizing hormone (LH), 2.60 mIU/ml; follicle-stimulating hormone (FSH), 4.57 mIU/ml; adrenocorticotropic hormone (ACTH), 37.4 pg/ml; thyroid-stimulating hormone (TSH), 2.68 μIU/ml). T1-weighted magnetic resonance (MR) imaging with contrast (gadolinium) showed homogeneous tumor enhancement, but adjacent tissue was not edematous in T2-weighted views. The mass did not regress, despite a 4-week course of the dopamine analog, cabergoline (1 mg/week), and serum PRL level (2207 ng/ml) remained elevated. Relative to status at admission, the right lateral ventricle also had enlarged somewhat, due to obstruction at foramen of Monro (Fig. 1b). In light of this intransigence, surgical debulking of tumor was elected first, through craniotomy and then via transsphenoidal approach. The hardened elastic quality of the tumor and its strong attachments to neighboring structures prevented complete removal, but related mass effect was significantly reduced and serum PRL level declined (to 250 ng/ml) in steps at each procedural stage. But the normalization of PRL level had not been achieved with the administration of cabergoline increased up to 0.5 mg/day. The residual tumor showed no evidence of regrowth or distant metastasis 1 year after surgery.

**Fig. 2:**
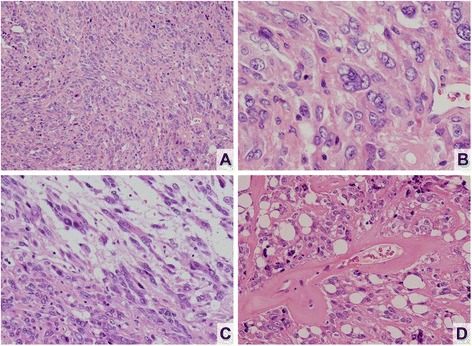
H&E staining of prolactinoma. Pleomorphic ovoid tumor cells in dense and disorderly proliferation (**a**); atypical multinucleated giant cells and prominent nucleoli seen in higher magnification (**b**); proliferation of spindle cells with elongated nuclei and prominent nucleoli (**c**); hyalinized vessel walls as frequent finding (**d**)

**Fig. 3:**
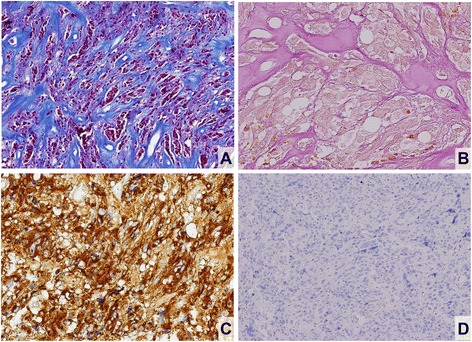
Immunohistochemistry of prolactinoma. Conspicuous fibrous change in tumor confirmed by Masson trichrome stain (**a**); Pearse’s PAS stain verifying chromophobic tumor cells (**b**); strong reactivity of tumor cells for PRL, with paranuclear localization (**c**); tumor cells negative for S-100 protein (**d**)

Histopathologically, hematoxylin and eosin (H&E)-stained sections of specimens from the first and second surgical procedures similarly were composed of polygonal and spindle cells with round, oval, or elongated nuclei and eosinophilic cytoplasm (Fig. 2a–c). The cells formed irregular nests or short fascicles, accompanied by frequent hyaline changes in vessel walls and vacuolation (Fig. 2d). Some nuclear pleomorphism was prominent; on the other hand, neither a high mitotic rate nor necrosis was evident (Fig. 2b, c). Conspicuous fibrous change of tumor stroma was noted focally as well (Fig. 3a). The chromophobic nature of tumor cells was confirmed by Pearse’s Periodic Acid Schiff (PAS) stain (Fig. 3b). Immunostains showed strong diffuse positivity for PRL (Fig. 3c), chromogranin-A, and synaptophysin. No other hormones (GH, TSH, ACTH, LH, FSH) were expressed by tumor cells, and the other markers, including S-100 protein (Fig. 3d), glial fibrillary acidic protein (GFAP), epithelial membrane antigen (EMA), cytokeratin AE1/AE3 (CK AE1/AE3), vimentin, p53, and bcl-2, were also negative by immunostaining. Ki-67 labeling index was approximately 2 %.

A final diagnosis of PRL-producing pituitary adenoma was reached after considering the immunohistochemical profile, suprasellar location, and prolactin production of the tumor, as well as the absence of metastatic foci and invasion into surrounding tissue.

### Discussion

The histopathologic traits of this tumor were highly unusual. Uniform, rounded, or polygonal cells with round-to-oval nuclei are typically seen in pituitary adenomas. In ordinary pituitary adenomas, cells are arranged in a perisinusoidal fashion, and mitotic figures or necrotic cells are rare. However, tumor morphology is known to change following dopamine agonist (DA) treatment. Moritetsu et al. reported necrotic changes in prolactinomas after long-term administration of bromocriptine, also identifying acellular areas (from tumor involution) where hyaline deposition and fibrosis were variably seen and describing visible tumor atypism (hyperchromatic or giant nuclei) [1]. After short-term exposure to DA, apoptotic cells/bodies [2] and multinucleated giant cells may be present [3], but fibrous change is exceptional. In those instances where use of DA has altered tumor morphology, therapeutic efficacy is commonly signaled by tumor involution and/or decline in serum PRL level, plus diminished PRL immunoreactivity (especially in the small cells that populate prolactinomas) [4]. In the patient reported here, broad fascicular fibrosis of tumor was focally conspicuous, and overt cellular atypia was observed, even though a relatively short course (4 weeks) of medical therapy was given preoperatively. Considering that the serum PRL level on admission was lowered by nearly one half through medication, the effects of DA may account for some of these manifestations. However, PRL immunoreactivity persisted postoperatively, without apoptotic changes or necrosis, none of this in keeping with response to DA. The stromal fibrosis may possibly reflect the longer period of growth and greater size of this lesion. There have been sporadic reports of pituitary adenomas that histologically show cellular atypia and bizarre nuclei. Matsumoto et al. have also documented a growth-hormone-producing giant pituitary adenoma with marked cellular atypia, and Moriwaki et al. have a recurrent pituitary adenoma harboring huge, bizarre nuclei [5].

In instances where an intracranial mass and hyperprolactinemia coexist, dysfunction of the hypophyseal stalk due to the mass effect of various tumors (i.e., chordoma, schwannoma, paraganglioma, or mixed germ cell tumor) [6] and tumors arising near sella, other than prolactinoma, must be considered. Differential diagnosis was required since the histological findings in this case were different from that of typical pituitary adenoma. In particular, the proliferation of spindle cells was striking, implicating a host of diagnostic possibilities (see Table 1). Spindle cell oncocytoma (SCO) contains fascicles of eosinophilic spindle cells with granular cytoplasm, and its immunophenotype is distinctive (positive for vimentin, EMA, and S-100; negative for pituitary hormones, synaptophysin, chromogranin, and cytokeratins) [7]. Pituicytoma consists of elongated, bipolar spindle cells in interlacing fascicles or storiform array, with variable immunopositivity for vimentin, S-100 protein, and GFAP [7]. Paragangliomas regularly exhibit nested patterns (Zellballen configuration) and are rimmed by sustentacular cells. The latter generally are immunopositive for GFAP, chromogranin-A, and neuron-specific enolase, and sustentacular cells are positive for S-100 protein [8].

**Table 1:** Histology and immunostaining in differential diagnosis of tumors

Staining	Case presentation	Pituitary adenoma	Pituicytoma	Paraganglioma	Spindle cell oncocytoma	Meningioma	Granular cell tumor
Histology	See text and Fig. 2	Monotonous arrays of chromophobic cells in diffuse, papillary, or sinusoidal proliferation	Variably lobulated sheets of cells with copious cytoplasm and round, uniform nuclei with small nucleoli	Nested pattern (Zellballen configuration) and rimmed by sustentacular cells	Eosinophilic spindle-shaped or polygonal cells with granular cytoplasm (abundant mitochondria)	Spindle or polygonal cells with large oval nuclei and indistinct cell borders	Abundant granular eosinophilic cytoplasm with round monomorphic nuclei
Pituitary hormones	±	±	−	−	−	−	−
Cytokeratin	−	±	−	−	−	5 %	−
EMA	−	>50 %	−	−	+	+	−
S-100 protein	−	−	+	+	+	20 %	+
Vimentin	−	−	−	±	+	+	+
GFAP	−	−	+	±	−	−	±
Synaptophysin	+	+	−	+	−	−	−
Chromogranin-A	+	90 %	−	95 %	−	−	−

*EMA* epithelial membrane antigen, *GFAP* glial fibrillary acidic protein

This patient’s tumor cells were giant atypical nuclei, giving the impression of malignancy. Pituitary carcinomas do not always show nuclear atypia, pleomorphism, necrosis, and hemorrhage as conventional indicators of malignancy, but mitotic count or Ki-67 labeling index is apt to be increased, with a clear tendency for cerebrospinal or systemic metastases. In this case, the tumor cells were proven chromophobic by Pearse’s PAS stain, showing immunopositivity for PRL, chromogranin-A, and synaptophysin but no immunoreactivity to S-100 protein, vimentin, and GFAP. Mitotic figures were scarce in tissue samples, and the Ki-67 labeling index was approximately 2 %. This immunohistochemical profile is quite compatible with pituitary adenoma. The details of antibodies used for immunohistochemical study are summarized in Table 2.

**Table 2:** Antibodies used for immunohistochemical study

Primary antibody	Source	Pretreatment	Category	Dilution
PRL	DAKO	None	Rabbit polyclonal	1:1500
GH	DAKO	None	Rabbit polyclonal	1:1500
FSH	DAKO	None	Rabbit polyclonal	1:100
ACTH	DAKO	None	Mouse monoclonal	1:2500
Cytokeratin	Leica BOND	Proteinase K	Mouse monoclonal	1:100
EMA	DAKO	Tris/EDTA pH 8	Mouse monoclonal	1:100
S-100 protein	Nichirei	None	Rabbit polyclonal	Predilute
Vimentin	Leica BOND	Tris/EDTA pH 8	Mouse monoclonal	1:200
GFAP	Leica BOND	Tris/EDTA pH 8	Mouse monoclonal	1:300
Synaptophysin	Leica BOND	Tris/EDTA pH 8	Mouse monoclonal	1:100
Chromogranin-A	DAKO	Tris/EDTA pH 8	Mouse monoclonal	1:400
bcl-2	DAKO	Tris/EDTA pH 8	Mouse monoclonal	1:1000
TP53	Leica BOND	Tris/EDTA pH 8	Mouse monoclonal	1:40
Ki-67	DAKO	Tris/EDTA pH 8	Mouse monoclonal	1:400

## Conclusions

Herein, we describe a patient who underwent treatment for a sizeable and visibly atypical prolactinoma. Similar to PRL-producing adenomas, sellar tumors also may exhibit hyperprolactinemia. Furthermore, DA administration can cause secondary changes in the histological appearance of the tumor. Thus, histopathological diagnosis can be extremely challenging in cases of PRL-producing adenomas characterized by remarkable spindle-shaped cells with nuclear pleomorphism, as described in the present study.

## Consent

Written informed consent was obtained from the patient for publication of this case report and the accompanying images. A copy of the written consent is available for review by the editor-in-chief of this medical journal.

## Abbreviations

ACTH: adrenocorticotropic hormone
DA: dopamine agonist
EMA: epithelial membrane antigen
FSH: follicle-stimulating hormone
GFAP: glial fibrillary acidic protein
GH: growth hormone
LH: luteinizing hormone
PRL: prolactin
TSH: thyroid-stimulating hormone
